# Hybrid Hydroxyapatite–Metal Complex Materials Derived from Amino Acids and Nucleobases

**DOI:** 10.3390/molecules29184479

**Published:** 2024-09-20

**Authors:** Alondra Jiménez-Pérez, Marta Martínez-Alonso, Javier García-Tojal

**Affiliations:** Departamento de Química, Facultad de Ciencias, Universidad de Burgos, Plaza Misael Bañuelos s/n, 09001 Burgos, Spain; alondrajp@ubu.es (A.J.-P.); mmalonso@ubu.es (M.M.-A.)

**Keywords:** amino acid, calcium phosphate, hybrid material, hydroxyapatite, nucleobase, nucleic acid, peptide, protein

## Abstract

Calcium phosphates (CaPs) and their substituted derivatives encompass a large number of compounds with a vast presence in nature that have aroused a great interest for decades. In particular, hydroxyapatite (HAp, Ca_10_(OH)_2_(PO_4_)_6_) is the most abundant CaP mineral and is significant in the biological world, at least in part due to being a major compound in bones and teeth. HAp exhibits excellent properties, such as safety, stability, hardness, biocompatibility, and osteoconductivity, among others. Even some of its drawbacks, such as its fragility, can be redirected thanks to another essential feature: its great versatility. This is based on the compound’s tendency to undergo substitutions of its constituent ions and to incorporate or anchor new molecules on its surface and pores. Thus, its affinity for biomolecules makes it an optimal compound for multiple applications, mainly, but not only, in biological and biomedical fields. The present review provides a chemical and structural context to explain the affinity of HAp for biomolecules such as proteins and nucleic acids to generate hybrid materials. A size-dependent criterium of increasing complexity is applied, ranging from amino acids/nucleobases to the corresponding macromolecules. The incorporation of metal ions or metal complexes into these functionalized compounds is also discussed.

## 1. Introduction

The geological relevance of phosphates is considerable. They are found in several formations, such as fossils and about 200 groups of minerals present in igneous and sedimentary phosphate rocks (PRs) [1,2,3,4]. In particular, calcium phosphates (CaPs) have gained attention due to both their geological relevance as primary phosphorus ores and their biological roles. The abundance of apatite-type minerals (chloro-, fluor-, and hydroxy-apatite), with the formulae Ca_10_(Cl,F,OH)_2_(PO_4_)_6_, make them stand out as ores from a mining point of view. Other prominent examples of CaP minerals are brushite CaHPO_4_∙2H_2_O, dahlite 3Ca_3_(PO_4_)_2_∙CaCO_3_, monetite CaHPO_4_, and whitlockite Ca_9_Mg(HPO_4_)(PO_4_)_6_.

Nevertheless, many other CaPs, apart from the above-mentioned mineral phases, have been studied in depth [5]. Their formulae usually exhibit atomic Ca/P ratios between 0.5 and 2 (in general, the lower the Ca/P ratio, the more acidic and soluble the calcium phosphate phase). Table 1 shows some of the CaPs we will discuss in this work due to their involvement in the processes described here [6]. Nonetheless, the most common members of this family, according to their solubility products [7], are hydroxyapatite and the polymorphic forms of tricalcium phosphate (TCP). From now on, it must be emphasized that the formulae given in the present review follow the International Union of Pure and Applied Chemistry (IUPAC) recommendations for chemical nomenclature, but we have retained the traditional names given in the literature for a better understanding and connection with the sources [8].

Regarding the CaP-containing apatite Ca_10_X_2_(PO_4_)_6_ family, different minerals with a Ca/P = 1.67 molar ratio can be found depending on the X anion, such as fluorapatite (Ca_10_F_2_(PO_4_)_6_), carbonate–fluorapatite (Ca_10_F_2_(PO_4_,CO_3_)_6_) and chlorapatite (Ca_10_Cl_2_(PO_4_)_6_) [25]. They exhibit varying levels of crystallinity and solubility [26]. Amongst them, hydroxyapatite (HAp, Ca_10_(OH)_2_(PO_4_)_6_) is the most significant compound in the context of both biology and geology. In the living world, HAp is involved in calcification, which is the biological process of the deposition of calcium in tissues and body structures. In terms of weight, HAp is the main component of bones and teeth in vertebrates. In this sense, about 60–70% of bone [27,28] and even 96% of tooth enamel [29,30] is HAp, with a slight amount of replacement of the PO_4_^3−^/OH^−^ groups by anions, mainly CO_3_^2−^ (2–8%, generating the so-called *bioapatite* [31,32]).

**Figure 1:**
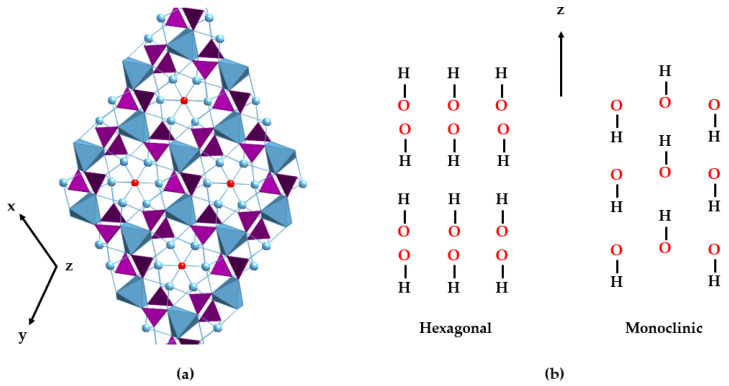
View of the hexagonal crystal structure of HAp (**a**). PO_4_^3−^ tetrahedra are represented in purple, the positions of the OH^−^ anions in red, and the two different sites for the Ca^2+^ ions in blue. The metal ions are depicted as balls (Ca2) and blue polyhedra (Ca1), respectively. (**b**) Schematic drawings of the hydroxide arrangements of hexagonal (left) and monoclinic (right) structures along the *z*-axis.

HAp can typically be found in two crystallographic forms [33,34]: the hexagonal and monoclinic phases (see Table 1). Stoichiometric HAp gives rise to the thermodynamically more stable monoclinic polymorph [35,36,37]. However, factors such as the inclusion of ions and the formation of vacancies cause it to take the hexagonal form, the most common phase in the mineral and biological worlds. In hexagonal HAp, hydroxide OH^−^ ions stack in channels along the [001] direction. The arrangement of tetrahedral phosphate ions distributes the calcium ions (Ca^2^⁺) in two different crystallographic sites. Ca1 is parallel to the *c*-axis and links to nine oxygen atoms from six different phosphate tetrahedra: three phosphate oxyanions act as monodentate ligands through the O1 oxygen atoms, and the other three phosphates behave as bidentate through the O2 and O3 sets of atoms. The Ca2 atoms are placed on the hexagonal screw axes, in polyhedra with an irregular sevenfold coordination whose environment contains one O1, one O2, and four O3 phosphate atoms and a hydroxide ion [38,39]. Differences with the monoclinic structure are subtle and mainly involve the orientation of hydroxide anions. In the hexagonal cell, two adjacent hydroxides point in the opposite direction, but in the monoclinic structure, all the hydroxides in a given column point in the same direction, which is reversed in the next column [40] (Figure 1).

HAp is a highly versatile material and, therefore, the field of applicability of this ceramic covers several areas. It has found use in biomedical research (implants and bone regeneration; scaffolds for tissue engineering and drug delivery), water purification (adsorbent for sequestering contaminants like heavy metals, dyes, and organic and inorganic pollutants, such as hydrocarbons and phosphates, and for reducing water hardness), batteries (energy storage), catalysis (catalysts, such as photocatalysts, or as active phase support), nanocarrier of biocides or elicitors in agriculture, ion exchange (membranes and filters for the separation and purification of liquids), and sensors (gas, temperature, biomolecule, ion, and pH sensors) [41,42,43,44,45].

**Figure 2:**
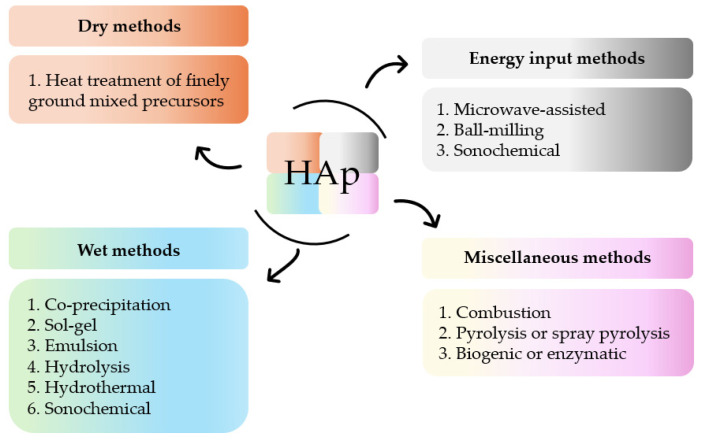
Summary of methods for the synthesis of HAp.

The versatility of HAp and its multiple applications can be tailored by a thorough control of certain design factors. These factors, which can be modified by different synthetic methods, are the reaction time, pressure, rate, nature and order of the precursor’s addition, concentration of the reactants, pH, and temperature [46,47,48,49]. The attained products show distinct composition (e.g., Ca/P ratio), particle size (powder, granular, macro- or microporous), degree of crystallinity, and morphology. Numerous synthetic routes to obtain polycrystalline HAp powder have been developed over the last decades. Post-synthesis parameters, such as re-immersion in solutions of the isolated solids or further thermal treatments, have also been analyzed [50]. All these procedures can be summarized in four different groups of methods (Figure 2) [51,52,53,54,55,56].

The morphology and crystal size of HAp depend on the crystal formation rate. The latter, in turn, is influenced by supersaturation, which correlates with the initial concentrations of calcium (i[Ca]) and phosphate (i[PO_4_]) ions, the so-called iCa/P ratio, and ipH of the solution [57]. In a study conducted by Szterner and Biernat [58], it was found that lower [Ca] levels (0.025 and 0.050 mol/dm^3^) produced HAp whiskers, whereas higher concentrations (0.1 and 0.2 mol/dm^3^) led to the formation of hexagonal rods. Furthermore, the crystallite size of HAp nanoparticles diminishes with decreasing [Ca]. These results suggest a significant influence of calcium ion concentration on the shape and size of HAp crystals during synthesis [59].

The relationship between the pH and shape of HAp crystals is crucial. The modification of the ipH value causes a change in the structure and morphology of the crystals, including spheres, rods, needles, wires, whiskers, spherulites, belts, etc. [60,61,62,63,64,65,66]. pH is a pivotal parameter to be considered for the solubility and dissolution of several ions in solution. Variations in this value generate different concentrations of OH^−^ and H_3_O^+^ ions, thereby inducing changes in Ca^2+^ and PO_4_^3−^ levels [67,68]. In the process of synthesizing HAp, incorporating a base like NH_4_OH leads to an elevated concentration of OH^−^ ions, consequently boosting the reaction’s alkalinity. This occurrence arises from the release of additional hydroxide ions, which can facilitate precipitation during synthesis. HAp is preferentially formed under neutral or alkaline conditions. Acidic conditions give rise to the formation of different phases [69,70]. Depending on the pH value, various crystalline phases are obtained, primarily due to the prevalence of the phosphate anion. In fact, H_2_PO_4_^−^ ions remain stable at pH levels between 3 and 6. If the pH increases between 8 and 11, HPO_4_^2−^ ions become predominant, but beyond pH 12, phosphate ions PO_4_^3−^ prevail [71]. Thus, the interplay between temperature, pH, and the order of the addition of precursors yields CaP precipitates, usually as mixtures of the OCP, DCPA, and HAp phases, where HAp tends to be the most abundant phase at pH > 5.3, whereas the former prevails at pH < 3.8 [72,73].

Temperature and pressure during synthesis also have a significant impact on the crystal growth of HAp [74], i.e., crystal size and shape. An optimal method for achieving pure and uniform HAp involves using high temperatures and pressures, as lower values result in the formation of diverse crystalline phases [58,66].

**Figure 3:**
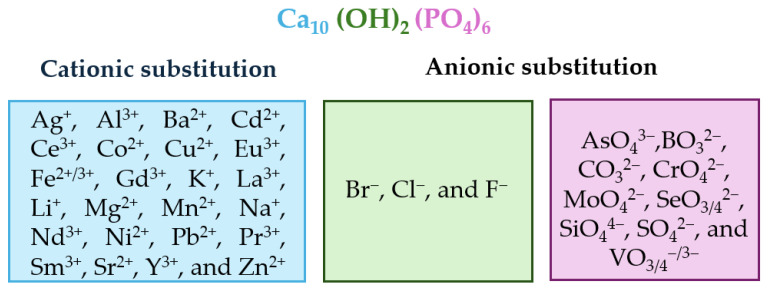
Alphabetically ordered ions in doped HAp, both cationic and anionic positions.

The chemical composition and vacancies present in the HAp crystal structure allow for a wide variety of anionic substitutions (involving hydroxide or phosphate groups) or cationic substitutions (like mono-, di-, and trivalent metals). The replacements cause disturbances in the crystalline network, which decreases crystallinity and therefore increases its capacity for reabsorption in physiological media. Ionic substitution in this ceramic material has gained importance in recent years, as summarized in Figure 3, which shows some of the replacements carried out [75,76,77,78,79,80,81,82,83,84].

The interaction of biologically active metal ion complexes with hard tissues, like bones or teeth, has propelled the study of the interplay between HAp and coordination compounds [85,86]. In some cases, an inhibition of HAp growth in the presence of a metal complex has been shown [87]. In addition, the search for HAp-containing multifunctional materials with new or improved properties has led to the fabrication of hybrid HAp–metal complexes as new steps in the process of achieving systems with increasing complexity. Thus, coordination compounds formed by transition metal ions linked to carboxylates, phosphonates, Schiff bases, or polypyridyl-type ligands, amongst others, have been incorporated into the inorganic HAp kernel [88,89,90,91,92,93,94,95,96]. A few years ago, Barbosa et al. wrote a very interesting review in this field [97]. Even though a deep bibliographic search on this matter falls outside the scope of the present review, the ligand behavior of most biomolecules is a nexus of these systems, including of the ternary HAp–biomolecule–metal systems that will be discussed later. This has prompted us to include the short insight given in this paragraph. Some HAp–biomolecule–metal compounds have been published recently. One of them incorporates glucose-6-phosphate and Cu(II)/Zn(II) ions into the HAp matrix, generating HAp-functionalized nanoparticles with good survivability and adhesion to osteoblast cells [98]. Another example links polydopamine to an alginate/Sr(II)/HAp composite, promoting osteogenic differentiation and vascularization [99]. In another case, β-cyclodextrin reacts with an inorganic zinc phosphate@HAp matrix, giving rise to a composite that serves as a nanocarrier for the antitumoral drug cisplatin [100].

Currently, various efforts are underway to achieve the synthesis of new materials from biomimetic and bio-inspired perspectives [30,101,102]. This search must take into account the features involved in the biomineralization processes, which are induced by physical and chemical factors, but also conditioned by the biological environment or presence of different living organisms [103,104,105,106,107,108]. In particular, research on innovative biomaterials aimed at bone tissue regeneration through the covalent functionalization of ceramic materials with biomolecules, or closely related models, has been fruitful [109,110,111]. Of course, CaPs are among the inorganic materials that are most likely to be used in bone healing [112,113,114,115]. But the relevance of these studies is also related to the dark side of the biomineralization process of HAp and other CaPs, which are involved in the formation of urinary stones and in vascular calcification, which can promote cardiovascular diseases [116,117]. The knowledge of the underlying mechanisms in both biologically essential and undesirable processes can be applied to different therapeutic strategies.

In the case of bone [118,119,120,121,122], mineralization occurs on the bone surface, where cells called *osteoblasts* secrete matrix structural proteins (mainly collagen, but also glycoproteins and proteoglycans) and the peptide hormone osteocalcin. The layers formed by type I collagen, microfibrils containing triple-helix collagen, act as templates in a complex process that has not yet been fully clarified. After a possible transient formation of ACP particles, the process leads to the deposition of HAp nanocrystals with platelet shapes and their *c*-axis aligned with the collagen fibrils. Hence, the mineralized collagen fibers constitute the structural units of bones [123,124]. In particular, the behavior of the inorganic matrix was explored by a combined Raman/^31^P-NMR study whose synthetic methodology provided a slow incorporation of base (NH_3_) from pH 2 to pH 10 [125]. The study found that the concentration of the tested biological components (a synthetic polyaspartate mimicking non-collagenous proteins, citrate, and collagen) could control the usual sequence of solid precipitates, represented in Equation (1), to reach the final crystalline HAp. Thus, polyaspartate stabilized OCP and citrate inhibited its formation and precluded HAp precipitation, whereas the great influence of collagen in the structure of HAp showed no dependence on the concentration.

(1) $$Solution\overset{0–96\;min}{\to }ACP\overset{96–480\;min}{\to }OCP\overset{480min–6days}{\to }HAp$$

As a result, bone tissue is produced. By weight, its average composition is approximately 65% inorganic components, 25% organic components, and 10% water. The main inorganic component is calcium-deficient carbonate hydroxyapatite (90%). In the case of the organic fraction, it is largely type I collagen (around 90%), non-collagenous proteins (like osteocalcin, osteopontin, bone sialoprotein, osteonectin, and SPARC, among others, 2.50–3.75%), together with small amounts of citrate (1.5–2.0%) and lipids (1–10%) [126,127,128,129].

In the case of teeth, three hard structures can be distinguished: dentin, cement, and enamel. The amount of inorganic apatite matrix in dentin and cement is similar to that in bones, 70 and 65% in weight, respectively, and the percentages of organic and water content are 20 and 10% in both cases. Nonetheless, the HAp content reaches nearly 95–97% in enamel [130,131,132], where amelogenin is the most abundant protein in the organic matrix [133].

The way to tackle the incorporation of biomolecules in the HAp bone/teeth matrix involves ensuring the effective immobilization of biomolecules on the surface of HAp. Thus, it requires high affinity and specificity with the ceramic moiety, achieving a precise immobilization at the desired site and ensuring the maintenance of biological activity [134]. In research on the functionalization of HAp with biomolecules, various synthetic approaches have been described [135]. Given that we will focus our review on the anchorage of peptides and nucleic acid derivatives on HAp, we will provide, in the following paragraphs, a very brief survey on the functionalization of HAp with carbohydrates, lipids, and vitamins.

- **The biodecoration of HAp with carbohydrates** has been achieved by directly and covalently bonding nanostructured apatite granules to various polysaccharides, like cellulose [136,137], chitosan [138], pectine [139], carrageenan [140], alginate [141], hyaluronic acid [142,143], and, very recently, acemannan mucopolysaccharide [144]. Additionally, monosaccharides such as D-glucose, D-galactose, and L-fructose are also utilized in this context [145,146].
- **The interaction of HAp with different carboxylic acids** has been extensively analyzed based on the affinity of Ca^2+^ ions for carboxylate groups, such as those present in aliphatic (propionic, malonic, glutaric, adipic, maleic, fumaric…), aromatic, polycarboxylic, and lactic/glycolic acid derivatives or even more complex carboxylate-containing organic compounds [147,148,149,150,151]. Studies have also included fatty acids and lipids, including stearic acid, ricinoleic acid, linoleic acid, and oleic acid, among others [152,153,154,155]. In fact, the presence of lipids promotes the precipitation of HAp [156], given, at least in part, that phospholipids trigger the in vivo transformation of OCP into HAp [157]. These phase transitions seem to be crucial in the early stages of bone biosynthesis, probably boosted by the formation of calcium–phospholipid complexes [158].
- **The creation of hybrid materials of HAp matrices functionalized with vitamins,** such as folic acid [159] and biotin [160], has been described in the literature.

The aforementioned findings highlight the essential role that HAp plays in the construction of the hard tissues of vertebrates. Their organic components are mainly proteins. Because of this, the present review is focused on the study of synthetic HAp–biomolecule materials containing proteins, or their simplest constituents, such as amino acids and peptides. We have also included HAp–nucleic acid biomaterials and their nucleobase biological bricks, emphasizing the relevance of phosphate anions in many biological processes, including those involving energy or information transference. As far as we are aware, no neat reviews simultaneously covering both aspects—proteins and nucleic acids or their constituting bricks—have been published up to date. Notwithstanding this, perhaps the most interesting novelty of the present review are the sections dealing with the incorporation of metal complexes, which we have included at the end of each chapter.

**Figure 4:**
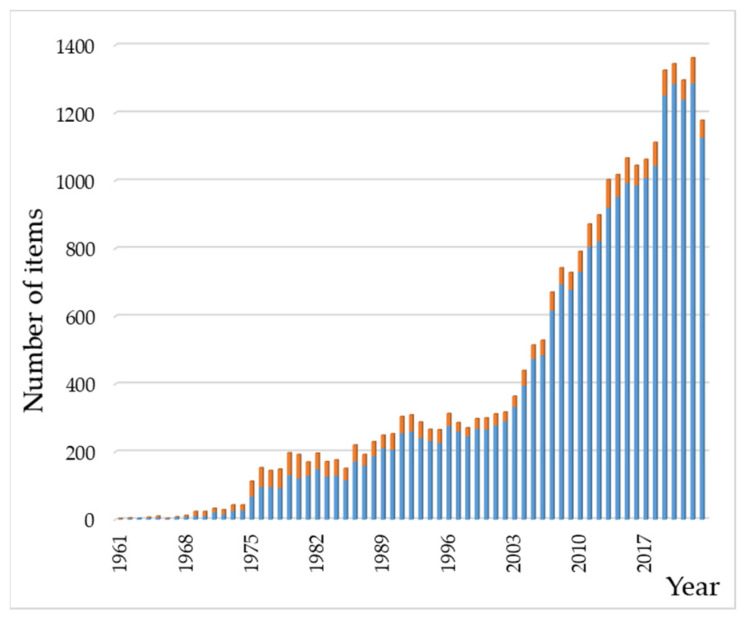
Chronological evolution of the number of entries in the bibliographic search on HAp–(AA–peptide–protein) (in blue) and HAp–(nucleobase/nucleotide/nucleic acid) (in orange) systems. The range of years covered in the figure is from 1961 to 2023. Data were collected from Web of Science (see main text).

A bibliographic insight into the scientific literature on these systems gives an impression of the cumulative efforts dedicated to them for years. With this purpose, we carried out a search in the Web of Science database using “hydroxyapatite” AND “(amino acid OR peptide OR protein)” as key terms present in abstracts. The search yielded 23,958 results [161]; the oldest paper was the early Cartier publication from 1948 [162]. In the same way, 2554 results were obtained for “hydroxyapatite” AND “(nucleic acid OR nucleotide OR nucleobase)”. Figure 4 depicts the results published from 1961 to 2023, showing a total of 23,569 and 2537 results for the peptide and nucleotide families, respectively. As can be seen, papers on HAp–protein related systems are much more numerous than those dealing with nucleic acid derivatives. Thus, this review is not an exhaustive description of the extensive bibliography about HAp–peptide–nucleotide systems but an essay aiming to introduce them. The perspective of this work is chemical, and it is mainly aimed at beginners or researchers who wish to have an initial and brief introduction to these hybrid systems.

## 2. Functionalization of HAp with Amino Acids, Peptides, Proteins, and Metal Complex Hybrids

### 2.1. HAp and Amino Acids

**Figure 5:**
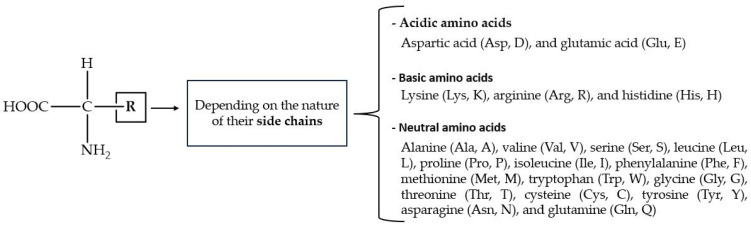
General structure of protein-forming AAs, with one- and three-character codes in parentheses.

Amino acids (AAs) have a wide range of biological functions. They are the building blocks of proteins and play essential roles in metabolism, enzymatic activity, cellular signaling, nitrogen balance, and detoxification [163]. The structural AAs inside proteins have a carboxyl (-COOH) fragment, an amino (-NH_2_) moiety, and organic R-substituents covalently bonded to the same carbon atom (Figure 5). Amino acids are classified as acidic, basic, or neutral, depending on the nature of their side chains. Out of the 20 known AAs with which cells form proteins, 9 cannot be synthesized by mammals (i.e., they are dietary essential).

Prebiotically relevant AAs, such as histidine, cysteine and arginine, have been reported to enhance phosphate adsorption onto iron oxy(hydroxide) minerals by ≈ 30% [164]. In the context of the interplay between AAs and phosphate ions, it can be expected that the arrangement of phosphate and calcium ions in the crystal structure of HAp [165] could favor their binding to AAs. In fact, the affinity between HAp and AAs rests on the presence of both carboxyl and amino functional groups in AAs, as well as in the side chain substituents (R) such as the -COOH (e.g., aspartic acid (Asp), glutamic acid (Glu)), -NH_2_ (e.g., lysine (Lys), arginine (Arg)), and -OH groups (e.g., serine (Ser), threonine (Thr)). These substituents are able to form covalent, ionic, and hydrogen bonds with the calcium and/or phosphate ions on the surface of HAp, affecting its structure and its physical, chemical, and biological properties.

Several preparative methods have been applied to obtain HAp-AA hybrid materials. Some of them involve direct synthesis in water under controlled conditions using solutions of soluble calcium and phosphate salts, such as Ca(NO_3_)_2_·4H_2_O and (NH_4_)_2_HPO_4_. The desired amino acid is added to the phosphate solution before adjusting the pH above 9 with NH_4_OH. Reactions are often carried out in a nitrogen atmosphere to avoid carbonation processes in such a basic medium. Note that carbonate ions are incorporated into the HAp structure by partial replacement of the OH^−^ (substitution in the A-sites) or PO_4_^3−^ ions (substitution in the B-sites) [166,167,168,169]. Finally, the hybrids are obtained as solids and, sometimes, further treatments (like moderate heating or hydrothermal) are performed [170]. Notwithstanding this, many other methods such as ultrasonic and microwave irradiation have been applied [171]. But regardless of the kinds of methods used, the experimental results unveil the complexity of the HAp/AA interaction. Studies show that complexes formed between charged AAs and Ca^2+^ and PO_4_^3−^ ions are time-dependent and react differently following pathways that depend on their level of organization and structure. Consequently, the time between the preparation of the solution of the precursor and their mix should be taken into account as an important variable in any biomineralization experiment [172].

The surface of HAp is neutral or slightly negatively charged in aqueous suspensions at neutral pH values. In the case of a basic pH, amino acids exist in their carboxylate ion (-COO^−^) form with a neutral amino group. Due to these negative charges, the electrostatic HAp-AA interaction could be considered weak because of the charge repulsion. Notwithstanding this, an investigation performed with the simplest AA (Gly) showed that the HAp–glycine (HAp-Gly) interaction modulated the morphology and size of the particles: the greater the amount of Gly, the smaller the size of the HAp-Gly crystals [173]. In other words, the crystallinity of HAp decreased with an increasing amount of Gly. And, more importantly, this result suggested that, for the interaction mechanism, the coordination of the amino acid to the calcium ions yields Gly-Ca metal complexes that participate in the formation of the crystal structure. Subsequent studies [174] have confirmed that the crystallinity of HAp diminishes in the presence of Gly. Moreover, the analysis of the structures suggests that Gly penetrates the inner channels, removing part of the OH^−^ anions from the *c* axis of the crystal lattice, and also links to the outer surface of the HAp when the AA is present in the medium from the beginning of the synthetic process at pH 12. However, when Gly is added to an aqueous suspension of a preformed HAp precipitate, only the surface vacancies are occupied by the amino acid.

The role of the AA in the above-mentioned HAp-Gly system is a useful introduction to the relationship between HAp-AA affinity and the size–morphology–solubility of the hybrids formed. This relationship affects the balance between HAp nucleation and crystal growth when different AAs are present in the medium. For instance, the effect of the addition of L-Lys and L-Tyr on the crystal growth of HAp showed an opposite behavior [175]. Whereas L-Lys provoked growth inhibition, the presence of L-Tyr improved crystal growth. Additionally, the presence of L-Asp resulted in a reduction in crystal size along the direction perpendicular to the *c*-axis, suggesting a preferential interaction of this AA with the crystal faces parallel to the *c*-axis [176]. A work on the affinities of Ala, Asp, Gly, Lys, and Ser to bioapatite (carbonate-containing HAp) at pH 9 showed the following affinity sequence (in decreasing order): Lys > Gly > Ala > Ser ≈ Asp. However, negatively charged amino acids (Asp and, partially, Ser) caused the greatest delay in the crystal growth of bioapatite and gave rise to the highest disorder degree along the long axis (the *c*-axis) [177]. In another study, the reaction of HAp with Ala, Arg, Gly, Lys, and Ser at pH 9 gave rise to positively charged solid particles, which evidenced the linkage between the negative AA carboxylate and the Ca^2+^ ions in HAp [178]. In addition, an increase in the size and hydrophobicity of the R-substituent in AA produced a decrease in the length of the particle, but it did not affect the width. In addition, the authors reported the formation of aggregated nanostructures by cross-linking in the Ala-HAp and Arg-HAp nanoparticles through in situ induced processes via thermal, hydrothermal, or chemical polymerization.

The adsorption that occurs between the amino acid and the HAp surface is reflected in the growth rate and follows a Langmuir-type behavior, providing information about the maximum adsorption capacity and affinity. Affinity constant (K_aff_) is a measure of the solute’s affinity for the surface of the adsorbent. A high affinity constant value implies prolonged adsorption. The affinity constants determined for the hybrid materials, calculated from the slope of the linear graphs according to Equation (2), are shown in Table 2 [179,180,181,182,183,184,185]. As a general trend, some of the studies conducted on the affinity of AA/HAp show how adsorption depends on the charge of the AA side groups (R-substituents), and the following sequence of increasing affinity is obtained: nonpolar < positively charged < polar < negatively charged.

(2) $$\frac{R_{0}}{R_{0}-R_{i}}=\frac{1}{\alpha }+\frac{1}{\alpha K_{aff}}\frac{1}{C_{eq}}\;$$

where *C_eq_*: amino acid concentration in solution in equilibrium with the HAp surface (mol/L). α: effectiveness factor. *R_i_* and *R*_0_: growth rates in the presence and in the absence of the additive. *K_aff_*: affinity constant (L/mol), equal to *k_ads_*/*k_des_* (specific rate constants for adsorption and desorption).

Therefore, charged AAs (positively or negatively) would have a stronger effect on inhibiting the precipitation of HAp by promoting nucleation processes. Molecular dynamic studies suggest the higher affinity shown by AAs containing charged residues could be due to the formation of superficial ion pairs and easy penetration into layers of water molecules on the HAp surface [187]. Additionally, it is important to consider geometric factors to determine the inhibitory effect of AAs on the growth of crystals. In the case of uncharged AAs, Table 2 shows that Tyr and Phe have the strongest K_aff_ and the strongest inhibitory effect on the growth of HAp particles. This is due to the presence of aromatic rings in their side groups, which possibly act as π-electron donors, resulting in a weak bond between the ring and the surface. However, affinity and growth control do not always run parallel. In the case of the negatively charged Asp and Glu, despite the higher affinity of Asp, adsorbed Glu covers a larger part of the crystal surface because it has a longer R group side chain compared to Asp’s corresponding side chain. Therefore, Glu is more effective in inhibiting HAp growth [188]. This conclusion has been supported by further studies, like the one performed by Dhayal et al. on Ala, Asn, Asp, and Glu [189]. Their work concluded that Glu-functionalized HAp showed the smallest particle size and asparagine (Asn)–HAp the largest, pointing out that smaller HAp nanoparticles were attained in the presence of charged polar AAs when compared to uncharged polar AAs. However, there is no total consensus on these affinity trends due to the different, sometimes even opposite, results published; e.g., the affinities reported by Jack et al. are greater for L-Lys than L-Asp [177]. It is probable that the experimental conditions drastically affect the results.

Neutral AAs, in particular Gly, Pro, and Hyp (4-hydroxyproline), are the main constituents of collagen, the most abundant protein in bones, close to 85–90% of the total protein content [190,191]. However, polar and charged amino acids are the main components in non-collagenous proteins (NCP, about 10–15% of protein in bone tissue) and play a crucial role in the mineralization of hydroxyapatite to build bone tissue. The role of AAs in the mineralization process of HAp has been reviewed by Tavafoghi and Cerruti in terms of the effects of AAs on nucleation, growth, morphology, and phase transformations, including in silico studies [186]. AAs can inhibit the mineralization of HAp by binding to its nuclei and blocking their growth. Thus, the incorporation of amino acids into nanocrystals allows for controlling the crystal dimensions, reducing the crystallinity of the apatite phase and promoting protein absorption and the proliferation and differentiation of osteoblasts, which are crucial for biomedical applications.

Specific interactions between the functional groups of amino acids and calcium ions on the surface of HAp are fundamental for these processes [192,193]. AAs interact with the surface through a combination of electrostatic interactions, hydrogen bonds, and direct coordination, influenced by the pH of the environment. However, it is not only the carboxyl and amino groups that play a relevant role in these interactions, but also the functional groups in the side chains of AAs, such as sulfate, carboxylate, imidazole, thiol, and hydroxyl. The latter can lead to quite different behaviors depending on the pH of the experiment. For example, Chauham and Singh reported the NCP-constituting L-His amino acid generated plate-shaped HAp crystals, regardless of the experimental conditions, while L-Glu was markedly influenced by the experimental conditions [194].

Certainly, at different pH values, the functional groups of amino acids can be protonated or deprotonated, affecting their ability to interact with the ceramic surface. At physiological pH (~7.4), carboxyl groups are generally deprotonated (-COO⁻) and amino groups can be protonated (-NH₃⁺), facilitating electrostatic interactions [195]. Specifically at physiological pH values, high adsorption rates (around 70%) were found for the interaction of Lys (polar basic AA) and Leu (nonpolar AA) with HAp, whereas the release did not exceed 12% [196], and zwitterionic AA forms were proposed. In particular, relatively fast kinetics have been reported for the adsorption of methionine onto non-stoichiometric and poorly crystalline HAp obtained at pH~7.4. In addition, it was found that the greater the HPO_4_^2−^ content, the higher the amount of amino acid adsorption [197].

On the other hand, at acidic pH, carboxyl groups can be protonated (-COOH), decreasing their capacity for electrostatic interaction with calcium ions. Interactions between AA and HAp mainly occur through COO⁻ groups, which act as ligands that coordinate to calcium ions. Notwithstanding this, it has also been reported that the amino group can interact with the surface through a hydrogen bond with water bridging, but this linkage seems to be less favorable [198,199]. In fact, amino groups have been proposed to be crucial for the binding of basic proteins to HAp, but not acidic ones. Moreover, replacement of -NH_3_^+^ by guanidinium was found not to affect the elution behavior of proteins adsorbed to HAp, which suggests that positive charge, and not the chemical nature of the group, is the key factor for the binding [200]. In the same way, the amino-containing AAs Lys and Arg generate a positively charged surface on HAp that improves the linkage to the negatively charged bovine serum albumin (BSA) protein, as will be discussed later in detail [201]. The negative pSer (ortho-phospho-L-serine) reduces protein adsorption. In this line of research, cyclodextrin-complexed HAp nanoparticles functionalized with tryptophan yielded good results in selective immunoglobulin removal, which makes these hybrids promising for plasma perfusion applications [202]. HAp modified with the acidic amino acid Asp was also shown to have selective loading capacity for basic (positively charged) proteins, such as cytochrome c and BSA [203].

In summary, the affinity between AA and HAp is related to the inhibitory effect on the nucleation and growth of HAp, which in turn depends on electrostatic interactions, hydrogen bonding, and the stability of the complexes formed between the AAs and the ions on the material’s surface. The adsorption of amino acids on surfaces is weaker compared to that of proteins due to their lower capacity for multiple bond formation. It means that a protein could bind a single HAp particle at different sites or/and more than one HAp particle.

### 2.2. HAp and Peptides

**Figure 6:**
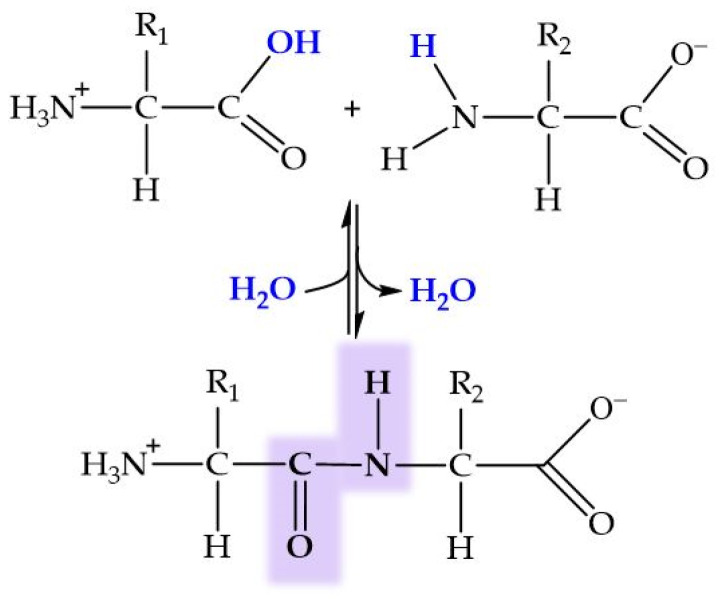
General structure of dipeptide and peptide bond formation by condensation. In blue, ions forming the water molecule.

Peptides are biomolecules formed by the linkage of 2 to 50 amino acids through peptide bonds. This type of covalent bond arises from a condensation reaction between the carboxyl group of one amino acid and the amino group of another, releasing a molecule of water in the process (Figure 6). Amide bond (peptide bond) formation in biological systems requires the presence of enzymes because of the unfavorable thermodynamic balance of the dehydration process in aqueous solutions. An exception to this generality is the recently reported dipeptide formation detected in the water–air interface of microdroplets [204].

Peptides fulfill a variety of essential biological functions in the human body [205,206,207,208]. They act as hormonal regulators and play crucial roles as neurotransmitters in the nervous system [209]. Additionally, peptides exhibit antimicrobial properties and are essential for the regulation of cell growth and development, as well as for facilitating protein digestion in the gastrointestinal tract. Furthermore, they operate as chemical mediators in cell communication, participating in the regulation of various physiological processes [210,211]. Peptides have been tested in organic/inorganic nanohybrids for biomedical applications [212]. Biomimetic mineralization through the self-assembling of peptides has been studied in depth [213]. In this context, the surface of HAp provides a specific environment that can be utilized to induce the binding of charged peptides, thus stabilizing the selected conformation. The functionality of most peptides heavily relies on their capacity to adopt a folded bioactive conformation. Hence, understanding the connections between HAp–peptide interactions and the clearly delineated secondary structure of peptides is crucial. Utilizing peptides to functionalize HAp, i.e., acting as hydroxyapatite-binding peptides (HBPs), represents a promising way for improving the cellular response during the process of bone tissue regeneration [214].

In their research on drugs against heart failure, Miragoli et al. [215] reported the therapeutic potential of CaP nanoparticles (NCaPs) as biocompatible and biodegradable inhalant nanocarriers for peptide delivery. They achieved the functionalization of the CaP surfaces with a mimetic peptide (MP = DQRPDREAPRS) and hemagglutinin scramble peptide (HA = YPYDVPDYA), whose negative charges suggested affinity for Ca^2+^ ions through a method involving a basic environment (pH 8.5). The presence of citrate as stabilizing agent allowed a negative charge in the NCaPs and modulated the particle growth. A further investigation was reported on functionalization of CaP nanoparticles (CaP-NPs) by the cyclic polypeptide antibiotic colistin (Col) [216], reaching a payload of about 80 mg Col per g CaP-NPs. The hybrid compound thus formed exhibited low cytotoxicity against pulmonary cells and retained the therapeutic potency of Col against the bacterium *Pseudomonas aeruginosa* (*P. aeruginosa*), becoming a promising substance for the treatment of cystic fibrosis.

Capriotti et al. [217] designed the peptide JAK1, composed of 36 residues, capable of folding into an α-helix on the surface of HAp. This peptide includes side chains of six γ-carboxyglutamic acid (Gla) residues, whose negative charges keep the peptide unfolded in the absence of calcium, thus preventing aggregation. In the presence of calcium, however, either in solution or on the HAp surface, JAK1 acts as a bidentate ligand to form Ca-O bonds and folds into an α-helix structure.

The affinity of JAK1 toward HAp was determined by fitting the adsorption isotherm data to a linearized form of the Langmuir equation. In surface studies, the dissociation constant (*K_d_*) quantifies the desorption tendency, in accordance with the equilibrium given in Equation (3).

(3) JAK1Adsorbed HApsurfaceSite+JAK1Solution←→Cm=CM+KdM

where *C*: concentration of the peptide in solution in equilibrium with the HAp surface (mol/L). *m*: amount of peptide bound to the surface as a function of peptide concentration (mol/gHA). *M*: maximum coverage of the peptide or protein possible per gram of HAp (mol/gHAp). *K_d_*: dissociation constant (mol/L).

The *K_d_* value for JAK1 adsorbed onto the HAp surface was *K_d_* = 310 ± 160 nM. This value implies that the peptide exhibited a much stronger binding affinity than the polyglutamic acid Glu_7_-Pro-Arg-Gly-Asp-Thr peptide studied by Fujisawa et al. [218], whose *K_d_* value on the HA surface was nearly 50-fold greater than Capprioti’s (*K_d_* = 13,500 ± 1500 nM).

Another study presented by Ling et al. [219] discusses new peptides, created and selected by phage display techniques, for developing new HAp–peptide hybrids as models of HBPs. Phage display is, basically, a methodological strategy [220] that uses standard recombinant DNA technology to insert foreign DNA fragments into filamentous phage genes that encode different phage coat proteins. It allows for the creation of new peptides and proteins that incorporate new amino acid sequences into the virion, which retains infectivity and displays the new peptide in a form accessible to specific antibodies [221,222]. Ling’s research group selected four new peptides, created and identified by phage display as possible HBPs [223,224]. Each peptide consisted of 12 units for their subsequent binding to HAp. The studied peptides were as follows: (1) VTKHLNQISQSY (VTK with a lysine (Lys) residue); (2) IYQSKHTLSNQY (VTK_s with the primary VTK sequence scrambled but retaining the same net charge); (3) VTKHLNEISQSY (VTK_7E, neutral charge by mutating a glutamine (Gln) residue to glutamic acid (Glu)), chosen to better understand the effect of AA composition on these peptides; and, finally, (4) VTKHLNQIS_P_QS_P_Y (pVTK, a variant of VTK with phosphorylated serine (pSer) residues, resulting in a net charge −3). These peptides had few acidic residues and a low charge density. They mainly consisted of hydrophilic amino acid side chain residues, among which Lys, Glu, and pSer are charged, while asparagine (Asn) and serine (Ser) are neutral and polar amino acid residues.

The peptides exhibited different conformational preferences when interacting with the ceramic surface. Helical structures were obtained with two peptides (VTK and VTK_7E), while the other two formed random coils (VTK_s and pVTK). Moreover, the binding affinity of the peptides to HAp was evaluated using Langmuir adsorption isotherms, and the resulting K_aff_ values are presented in Table 3. The binding affinities of the peptides were found to follow the sequence VTK_7E < VTK_s < VTK ≪ pVTK, revealing that the magnitude of the net charge of the peptide, rather than the number of charged side chain residues, determined the binding affinity. Further studies on pellets of HAp functionalized with pVTK showed the high effectivity of these pellets in inducing precipitation of bone-like apatite after immersion into a McCoy culture medium. The latter is a body fluid simulator with the ability to induce bone-like apatite precipitation on bioactive surfaces [225].

Co-precipitation in a single vessel is a versatile and efficient technique for materials synthesis, allowing for control over the composition, structure, and properties of the resulting material. In the study by Drouet, Subra, et al. [226], the authors carried out a one-pot synthesis of hybrid HAp–peptide nanoparticles through direct reaction of Ca(NO_3_)_2_∙4H_2_O, (NH_4_)_2_HPO_4_, and previously prepared phosphonated polyethylene glycol (P(PEG)) peptide conjugates at pH 9–10. For this purpose, they used two peptide sequences derived from fibronectin and laminin relevant to the promotion of cell adhesion and proliferation. The synthetic pathway included several previous stages, such as the preparation of P(PEG) peptide conjugates and peptide sequences. The peptide sequences Ac-LRGDNK-NH_2_ and H-GDPGYIGSR-NH_2_, referred to as RGD and YIGSR, respectively, were synthesized on resin using the Fmoc/^t^Bu method [227]. These compounds were coupled to form the desired conjugates, and the protecting groups were later removed to obtain the final products PO_3_H_2_CH_2_CH_2_CO-PEG3K-peptide (P(PEG3K)-peptide) and PO_3_H_2_CH_2_CH_2_CO-PEG3K-NEt_2_ (P(PEG3K)-NEt_2_). Subsequently, the apatite phase was precipitated in the presence of these stabilizing conjugates, adjusting the conditions to obtain stable colloidal suspensions. Finally, the nanoparticles were purified by dialysis to remove unreacted ions or organic molecules. The resulting hybrid nanoparticles exhibited a controlled size distribution, with the organic stabilizers anchored through the phosphonate terminal group. Thus, the PEG conjugates played a double role as stabilizing agents and bioactive moieties, controlling the size of the HAp–peptide nanoparticles.

Peptides influence various stages of CaPs’ crystallization, from dissolution to crystal growth, which is crucial for bone mineralization. Studies on peptides supported on ceramic matrixes show certain trends that point to the same objective. The charge density of the peptide is the primary determinant of its binding affinity and its ability to direct the nucleation and growth of HAp. The amphiphilicity of a peptide significantly influences its propensity to fold on a surface [228]. However, the secondary structure of the peptide, which can restrict the orientation of the side chain towards the surface, has a lesser impact on binding affinity to the surface in comparison. Additionally, this binding affinity is significantly increased in the presence of basic or acidic residues or peptide phosphorylation (even up to 10 times the peptide adsorption). Peptide adsorption to HAp surfaces occurs through interactions of charged and neutral polar residues with ions present on the ceramic surface. Moreover, stabilizers are essential for the synthesis and stability of NPs, allowing for control over their size and distribution. These studies concluded that the smaller the amount of stabilizer, the larger the nanoparticles, due to the minimization of steric hindrance.

Recently, the bone-targeting ability of poly(glutamic) acids (PGAs) with different chain lengths was explored [229]. The results showed that shorter PGAs favored in vivo bone targeting. The in vitro behavior was found to be the opposite: larger PGAs exhibited greater HAp affinity. These divergences seemed to be due to the rapid macrophage clearance of large PGAs, which limited their targeting ability in vivo.

### 2.3. HAp and Proteins

Proteins are macromolecules composed of amino acid chains connected by peptide bonds. They perform crucial functions for the proper functioning of the organism and can be classified into various types based on their functions and structures. Among them, we can find enzymes (which catalyze essential biochemical reactions), transport (hemoglobin), storage (ferritin), motor (myosin), defense (antibodies), hormonal (insulin), and structural proteins like collagen (Clg) and keratin, which provide support and shape to cells and tissues.

For decades, the affinity between HAp and proteins or peptides has been explored for separation purposes in the field of chromatography. Proteins with phosphate groups in their structure (phosphoproteins) exhibit a higher affinity, resulting in stronger binding to the HAp surface compared to non-phosphorylated proteins. In non-phosphorylated proteins, binding to HAp occurs through the negatively charged carboxyl groups interacting with the surface calcium ions. However, other sources of phosphate groups in proteins, such as phospholipids in lipoproteins, must be taken into account, [230,231]. In addition, the water molecules and ions present on the HAp surface, forming the hydration and the non-apatite layers, respectively, affect the immobilization of proteins on the particle [232].

This review will cover non-collagenous proteins (NCPs) in a broad manner but will primarily focus on the role of Clg in their linkage to the inorganic CaP matrix, as collagen represents the most abundant protein in mammals, at around 20–30% of the protein weight [233].

**Figure 7:**
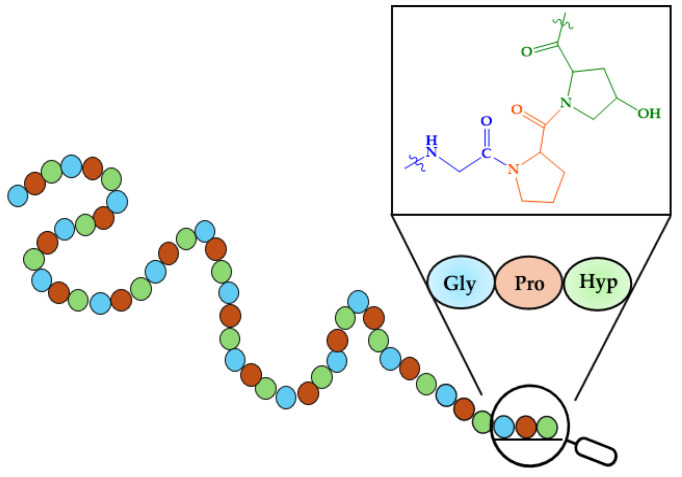
Primary structure of collagen.

As mentioned in the Introduction, bones are composed of 65–70% minerals and 30–35% organic molecules, predominantly type I collagen [234]. Type I collagen is characterized by its high content of proline, glycine, and hydroxyproline, which together represent over 50% of the amino acid composition, often arranged as Gly-X-Y repeats (where X and Y are Pro or Hyp). These amino acids assemble into Clg fibrils forming the fundamental triple-helix secondary structure. A representation of the primary structure of Clg is given in Figure 7.

The structural organization of this protein is relevant for the deposition of HAp within bone tissue. Type I collagen fibrils consist of tropocollagen as their basic unit, composed of three chains: two α-1 collagen chains (COL1A1) and one α-2 collagen chain (COL1A2) [235]. Tropocollagen has a diameter of 1.5 nm and a length of 300 nm. These tropocollagen units are aligned in an organized manner, and the regions between the fibrils, known as “hole zones”, are approximately 40 nm in length and 5 nm in width [236], exhibiting a high charge density, which is critical for mineral nucleation. These hole zones create favorable conditions for the accumulation of mineral ions, initiating the formation of incipient nuclei, which subsequently grow into larger, organized mineral structures. Furthermore, the size of these holes appears to limit inorganic growth. Bone sialoproteins, osteonectin, osteopontin, osteocalcin, and/or dentin matrix proteins—all NCPs—bind to tropocollagen and regulate the mineralization process of hydroxyapatite. These NCPs are rich in acidic amino acid residues or experience post-translational modifications that add acidic groups into their sequences, enhancing their affinity for Ca^2+^ ions. Within bone proteins, osteocalcin stands out for having a distinct amino acid residue that binds to calcium, γ-carboxyglutamic acid (Gla), so osteocalcin is also called bone Gla protein (BGP) [237].

Approximately 28 types of collagens have been identified, each sharing a triple-helix (TH) structure while differing in the composition of their α chains, thereby contributing to their distinct functional properties.

The five main types of collagens and their roles include the following [238,239,240,241,242]:

- Type I forms 90% of organic bone mass and is a major protein constituent in various tissues, such as tendons, ligaments, the cornea, or the skin.
- Type II is found in elastic cartilage, providing resilience and support to joints.
- Type III is present in muscles, arteries, and organs, offering structural support and elasticity.
- Type IV is located in skin layers, contributing to basement membranes and tissue organization.
- Type V is found in the cornea of the eye, some skin layers, hair, and placental tissue, contributing towards tissue stability and function.

Studies on the mineralization of apatite in the presence of protein suggest that collagen fibrils facilitate the formation of apatite from low [Ca^2+^] and [PO_4_^3−^] values [243]. Bradt et al. [244] described a wet method for the attainment of mineralized Clg gel by mixing an acid calcium-containing Clg solution with a buffered neutralized phosphate solution. This process allowed for the simultaneous assembly of collagen fibrils and precipitation of amorphous calcium phosphate (ACP), which subsequently transformed into crystalline HAp. The crystallization of HAp on the fibrils improved with the addition of polyaspartate, a synthetic polymer that mimics NCP function. Another approach studied by Qu et al. [245] employed type I collagen substrates immersed in a solution called simulated body fluid (SBF), which contained inorganic ions (from salts such as NaCl, NaHCO_3_, KH_2_PO_4_, MgCl_2_, and CaCl_2_) in similar concentrations to those in human blood plasma. The preformed substrates acted as templates for HAp deposition, gels on which minerals could nucleate and grow, mimicking the natural mineralization process in biological tissues like bones and teeth. The binding of Ca^2+^ to negatively charged carboxylate groups in collagen was found to be crucial in facilitating this nucleation [246]. Molecular dynamic simulations of the interaction mechanism of aspartic acid residues in collagen on the crystal HAp surface have revealed the impact of calcium vacancies on carboxylate adsorption and the stabilizing role of hydrogen phosphate in the process [247]. In addition, it has been proposed that HAp nucleation mainly occurs around charged amino acid residues in human type I collagen. In particular, arginine seems to play a pivotal role in the process. The thermodynamic barrier height for the formation of complexes with Glu and Asp with PO_4_^3−^ is higher than those with Lys and Arg [248]. Moreover, the phosphorylation (incorporation of negatively charged phosphate groups) of Clg nanofibers promoted the deposition of HAp [249].

Mesoporous hydroxyapatite nanoparticles coated with collagen have been successfully synthesized for the selective delivery of drugs to cancer cells [250]. These NPs exhibited biocompatibility, nontoxicity, and anti-inflammatory properties, highlighting their potential for future biomedical applications.

Regarding research on non-collagenous proteins, it has been demonstrated that certain highly acidic NCPs, characterized by multiple phosphorylation sites [219] or an integrin-binding RGD (Arg-Gly-Asp) domain, can bind HAp through their acidic protein regions [251] and directly influence controlled mineralization [252]. Some NCPs used as linker molecules to functionalize the surface of HAp include bovine serum albumin (BSA) and a small integrin-binding ligand, N-linked glycoprotein (SIBLING). Proteins from the SIBLING family located on human chromosome 4q21 include dentin matrix protein 1 (DMP1), dentin sialophosphoprotein (DSPP), matrix extracellular phosphoglycoprotein (MEPE), osteopontin (OPN), and bone sialoprotein (BSP). BSP and OPN are found in higher concentrations in bone tissues, whereas DMP1, DSP, and DPP are predominantly found in dentin. These proteins are characterized by domains that mediate cell adhesion and can functionalize mineralization by inhibiting calcification. Despite their strong affinity for HAp crystal surfaces, their structural flexibility enables other parts of the molecule to interact with other proteins or facilitate cell binding through the exposed RGD integrin-binding domains. SIBLING proteins are characterized by a high content of acidic amino acids such as Asp and Glu, but also contain basic residues like Arg and Lys. Upon ionization, these residues, along with Gln, Gly, Pro, and Ser, are known disruptors of the protein structure, which may influence the long-term ordering of their main conformational stability. Additionally, the SIBLING protein family has been observed to stably bind to Clg fibrils.

The concentration of organic additives, like collagen, citrate, and a synthetic polyaspartate mimicking an NCP, influences HAp crystallization. This concentration was found to modify the sequence of HAp formation (usually, first ACP, afterwards OCP, and, finally, HAp) [253]. The most outstanding result in this work was that confinement thermodynamically drives HAp formation by slowing down the kinetics in the formation of CaP precursors.

Apart from bones, HAp is also present in teeth. As previously mentioned, amelogenin is the most abundant protein in enamel (about 90% of the protein content). There, this hydrophobic peptide self-assembles to form an extracellular matrix which acts as a template for the continuously growing HAp crystals, presumably through nanosphere formation via oligomers [254]. Habelitz et al. revealed that the enamel protein amelogenin adopts ribbon-like supramolecular structures that template the growth of highly oriented HAp nanofibers. The mechanism is regulated by enzymatic processing and interaction with acidic nonamelogenin proteins [255]. It is remarkable that very small variations in the amino acid primary sequence can drastically influence the biomineralization of HAp. Thus, the change in one amino acid, proline to threonine, within the sequence in amelogenin, gives rise to defective enamel; this is present in a group of congenital disorders known as *amelogenesis imperfecta* [256].

Regarding other proteins, Kollath et al. [201] explored methods to improve the adsorption capacity of HAp powder for proteins such as BSA. Under neutral pH conditions in an aqueous solution, HAp has a neutral or slightly negative charge, while BSA is negatively charged. Specific linker molecules (L-Arg, L-Lys, and O-phospho-L-Ser) were used as functionalizing agents to modify the charge of BSA. Thus, the electrostatic interactions on the surface of the crystalline material were altered, improving the attraction between the powder and the protein. The carboxyl group of these linker molecules can interact with Ca^2^⁺ ions present on the HAp surface through electrostatic reactions, leaving the amino group available to react with BSA. Additionally, the amino group can form a hydrogen bond with the HA surface via a water molecule, although this interaction is weaker. Lys and Arg increased protein adsorption, whereas phosphoserine reduced it. This increase seemed to be due to a change in the surface charge of apatite, making the positively charged residues more prominent after functionalization, thereby enhancing the adsorption of negatively charged BSA. A slight increase in the zeta potential also significantly boosted protein adsorption. The findings indicated that adsorption capacity can be controlled through different functionalization, depending on the specific protein–carrier pair under consideration.

Medicinal research on biomaterials, which includes the search for nanocarriers to be used as drug delivery systems, has successfully found good candidates in hybrid compounds formed by HAp functionalized with proteins such as keratin [257], collagen [258], BSA [259], and buttermilk proteins [260], among others. However, not only HAp, but other CaPs, too, have been used as bioceramics derived from collagen scaffolds, including β–TCP or a silicon-based bioactive glass composed of 60% SiO_2_, 36% CaO, and 4% P_2_O_5_ (mol%) to yield a SiO_2_–CaO–P_2_O_5_ network [261]. The use of CaPs as inorganic matrices for bone morphogenetic proteins (BNPs) has also been investigated and recently reviewed [262,263,264], together with the field of protein-based active coatings in biomedical materials [265]. In fact, the creation of a synthetic bi-functional fusion protein has allowed for the targeting and monitoring of HAp biomineralization processes [266].

### 2.4. HAp–Peptide–Metal Complex Hybrids

The interaction of functionalized HAp–peptide hybrids with metal ions and coordination compounds has been explored. For instance, the use of HAp for removing Cu(II) ions in solution was investigated in the presence of Gly and ligands (ethylenediamine, en, and ethylenediamine tetraacetic acid, EDTA). The study ratified that the ligand sequestration effect minimizes the incorporation of transition metal ions into the HAp matrix [267]. But Cu(II) ions have also been included into calcium-deficient hydroxyapatite/multi-(amino acid) copolymers, showing good capacity for angiogenesis and osteogenesis [268].

The interaction of Zn-substituted OCP and the corresponding Ca-deficient Zn-HAp derivative with AAs (Asp, Cys, Glu, His, and Lys) was analyzed by Suzuki et al. [269], who reported that the release of Zn^2+^ ions in the presence of AAs was larger in the case of Cys and His.

Hybrid Sm-doped fluoroapatites (Sm-FAp) functionalized with Asp, Glu, Gly, and His were tested as catalysts in the synthesis of 1,2,4-triazole from 2-nitrobenzaldehyde and thiosemicarbazide, highlighting the excellent yield of the Gly hybrid [270]. Fe-derivatives in the same fluoroapatite–AA system showed good catalytic activity in the synthesis of thio-triazole compounds, in particular in the attainment of 1,2,4-triazolidine-3-thione products [271]. Fluorescence in Cd-FAp-Glu/His hybrids revealed that fluorescence intensity enhanced with the amount of AA adsorbed [272]

In turn, Chrissantopoulos et al. [273] created a ternary organometallic–AA–HAp by reaction of titanocene with Gly/Ala and HAp, whose growth was found to be inhibited in the presence of the complex.

Li et al. captured phosphorylated peptides by means of a hybrid compound formed by a magnetic Fe-containing metal–organic framework (Fe-MOF built with benzene-1,3,5-tricarboxylic acid) and inorganic HAp nanowires [274].

In the case of proteins, the adsorption of myoglobin on the surface of HAp modified with Ni^2+^, Cu^2+^, and Zn^2+^ has been analyzed [275]. For their part, Kalidas and Sumathi [276] have recently reported the preparation of a scaffold for bone tissue engineering based on a gelatin/polyvinyl alcohol/silk fiber reinforced with Cu-substituted HAp, which showed antimicrobial activity, biocompatibility, strong mechanical strength, and promising osteostimulation clues. On the contrary, the presence of Pb^2+^ was found to inhibit the binding of osteocalcin to hydroxyapatite [277].

Not only complexes, but also metal nanoparticles have been linked to HAp-AA scaffolds, like the hydroxyapatite/gold nanoparticles/Arg nanocomposite designed by Vukomanović et al. [278], or viceversa, as in the case of the construction of a silica/HAp/gold nanoparticle assembly to be used in phage display techniques [279]. The opposite strategy allowed for the construction of Asp-capped gold nanoparticles to induce HAp crystallization [280]. From the same perspective, PEGylated, peptide-coated superparamagnetic iron oxide nanoparticles (SPIONs) were investigated to bind the HAp present, in small amounts, in diseased cardiovascular tissues, in order to detect atherosclerosis or aortic stenosis [281]. Finally, the use of silver nanoparticles in dentistry, which involves interactions with CaP–protein composites, has recently been reviewed [282].

## 3. Functionalization of HAp with Nucleobases, Nucleotides, Nucleic Acids, and Nucleic Acid–Metal Complex Hybrids

### 3.1. HAp and Nucleobases

**Figure 8:**
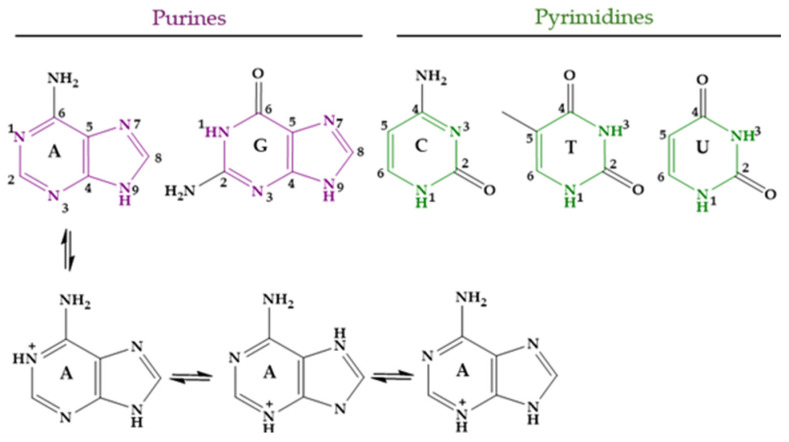
Nucleobases in their unprotonated form and the three lowest-energy tautomers of protonated Ade.

Nucleobases, or nitrogenous bases, are organic compounds that make up nucleotides, which are the essential building blocks of deoxyribonucleic acid (DNA) and ribonucleic acid (RNA). There are different nucleobases, but those likely found in DNA and RNA and thus involved in the genetic code are five: adenine (Ade, A), guanine (Gua, G), cytosine (Cyt, C), thymine (Thy, T), and uracil (Ura, U). Several tautomeric forms of them are possible. In the case of Ade, the three monocationic tautomers with the lowest energy are shown in Figure 8; one of them is protonated at N1 of the 9H tautomer of Ade, another at N3 of the 7H tautomer, and the last one at N3 of the 9H tautomer [283]. At physiological pH, approximately 7.4, the five main nucleobases exist almost completely in their keto and amino tautomeric forms. The corresponding pK_a_ values are shown in Table 4 [284,285,286,287].

The lack of functional groups to establish strong enough linkages with HAp precludes the direct binding of nucleobases to CaPs, so no papers dealing with this item have been found. Therein, the information given here must be placed in the context of the whole section.

### 3.2. HAp and Nucleotides

**Figure 9:**
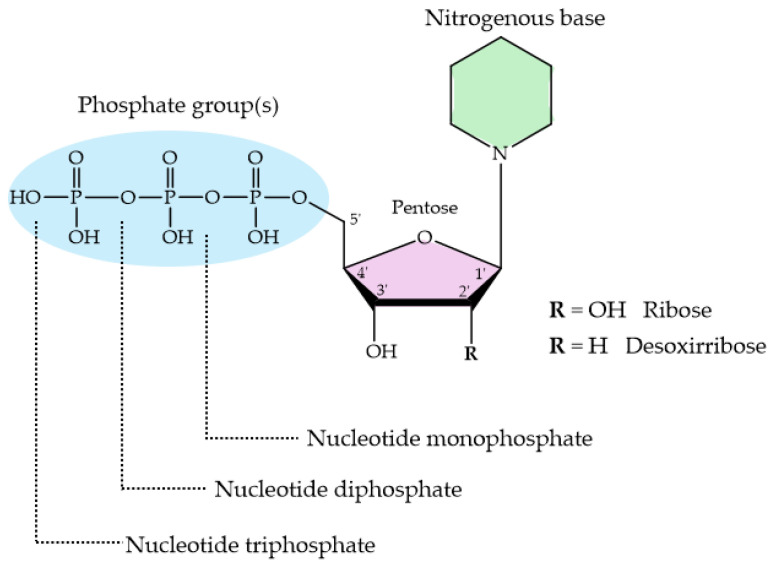
Basic structure of nucleotides with their three essential components. The nitrogenous base can be either a purine or a pyrimidine; the pentose sugar is either ribose or deoxyribose, and the phosphate group is attached to the 5′ carbon of the sugar.

Nucleotides are essential organic compounds that, arranged in an *anti* conformation, act as the monomeric constituents of nucleic acids. Nucleotides have a wide range of functions, serving as the primary energy currency in cells, as coenzymes or cofactors in enzymatic reactions, as second messengers in signal transduction, and playing a crucial role in the metabolism of carbohydrates, lipids, and proteins, etc. Figure 9 represents the basic nucleotide structure and the atom numbering. As can be seen, a nucleotide comprises three fundamental components: a nitrogenous base, pentose sugar (a five-carbon monosaccharide), and from one to three phosphate groups. Nucleotides can adopt numerous conformations in solution, engaging in rapid dynamic equilibrium [288].

The protonation sites in 2′- deoxynucleotides (dNXP^n^ where N = purine or pyrimidine; X = M, D and T; n = −2, −3 and −4, respectively) exhibit greater basicity compared to their ribose analogs (NXP^n^). The pK_a_ values of 2′-deoxyribose are more basic than those of ribose nucleotides. Table 5 shows the acid strengths for the different nucleotides: adenosine 5′-mono-, di-, and triphosphate (AMP^2−^, ADP^3−^, ATP^4−^); 2′-deoxyadenosine 5′-mono-, di-, and triphosphate (dAMP^2−^, dADP^3−^, dATP^4−^); guanosine 5′-mono-, di-, and triphosphate (GMP^2−^, GDP^3−^, GTP^4−^); 2′-deoxyguanosine 5′-mono-, di-, and triphosphate (dGMP^2−^, dGDP^3−^, dGTP^4−^); cytosine 5′-mono-, di-, and triphosphate (CMP^2−^, CDP^3−^, CTP^4−^); 2′-deoxycytosine 5′- mono-, di-, and triphosphate (dCMP^2−^, dCDP^3−^, dCTP^4−^); uridine 5′-mono-, di-, and triphosphate (UMP^2−^, UDP^3−^, UTP^4−^); and thymidine 5′-mono-, di-, and triphosphate (dTMP^2−^, dTDP^3−^, dTTP^4−^).

Taking H_2_(GMP) as an example, three deprotonation reactions can occur: from (a) N7H^+^ site; (b) PO_3_(OH)^−^ group; and c) N1H unit. Thus, in a general formulation, the deprotonation reactions for nucleoside 5′-mono-, di-, and triphosphates (NP^2−^/^3−^/^4−^) are described in Equations (4)–(6) [289], which yield the pK_a_ values given in Table 5.

(4a) $$H_{2}{\left(NP\right)}^{\pm /-/2-}⇌H{\left(NP\right)}^{-/2-/3-}+H^{+}$$

(4b) $$K_{H_{2}(NP)}^{H}=\frac{\left[H{\left(NP\right)}^{-/2-/3-}]{[H}^{+}\right]}{\left[H_{2}{\left(NP\right)}^{\pm /-/2-}\right]}\;$$

(5a) $$H{\left(NP\right)}^{-/2-/3-}⇌{NP}^{2-/3-/4-}+H^{+}$$

(5b) $$K_{H(NP)}^{H}=\frac{{[NP}^{2-/3-/4-}][H^{+}]}{\left[H{\left(NP\right)}^{-/2-/3-}\right]}$$

(6a) $${NP}^{2-/3-/4-}⇌{\left(NP-H\right)}^{3-/4-/5-}+H^{+}$$

where NP minus H means a N1H site of a guanine residue or the N3H site of a uracil/thymine residue loses its H.

(6b) $$K_{NP}^{H}=\frac{{\left[(NP-H\right)}^{3-/4-/5-}][H^{+}]}{\left[{NP}^{2-/3-/4-}\right]}$$

Depending on the specific nucleobase, only certain equilibria are applied. The nucleotides GXP and dGXP follow equilibria (4a), (5a), and (6a). For the nucleotides A/CXP and dA/CXP, only equilibria (4a) and (5a) are important, as they consider the deprotonation of the N1H^+^ site (4b) and the monoprotonated phosphate group (5b). For the nucleotides U/TXP and dU/TXP, it is only necessary to consider equilibria (5a) and (6a), which quantify the release of the proton from the monoprotonated phosphate residue and the deprotonation of the N3H site (6b).

An increase in basicity arises from the replacement of the 2′OH group with a 2′H atom, which diminishes the hydrophilicity of the nucleotide. As a result, the nucleotide solvation by water molecules is affected, leading to changes in its deprotonation behavior. Among all the nucleotides, the one with guanine is the most impacted, with the N7 site showing a notable increase in basicity, leading to a higher acidity constant. Consequently, the effect on the phosphate groups and N1H or N1H^+^ is smaller, but still present. These effects become more pronounced again only when the phosphate chain is long enough to form a macrochelate with N7, as in the case of triphosphates.

The bases of transphosphorylation, the exchange of phosphate groups, between nucleotides and HAp have been explored. In 1962, speculation began about the possibility of a structural relationship between nucleotides and the surface of apatite that could explain the accelerated transphosphorylation reaction found [299]. Several nucleosides di- and triphosphate were tested, together with inorganic pyrophosphate, in their reaction with different phosphates, including substituted apatites with Sr or Pb. The binding of nucleotides to apatites was found to be more efficient than that to other phosphates. In addition, the production of inorganic pyrophosphate in the reaction with nucleotides was detected. The terminal PO_4_^3−^ of the nucleotide was released to combine with a phosphate from the apatite crystal, forming P_2_O_7_^4−^. Further studies evidenced the role of P_2_O_7_^4−^ as an inhibitor of the biomineralization of HAp [300,301].

More recent studies have demonstrated that adsorption on the HAp matrix is affected by the charge of the nucleotide [302]. The presence of a cationic or neutral charge in a nucleotide results in absent or significantly reduced adsorption. Conversely, a net negative charge promotes significant chemical adsorption, which can be neutralized by acidification. Additionally, at neutral pH, it is known that the mere presence of phosphates in the nucleotide does not ensure adsorption on HAp. The number of phosphates can only influence the adsorption efficiency of a molecule if it carries an overall negative charge.

### 3.3. HAp and Nucleic Acids

**Figure 10:**
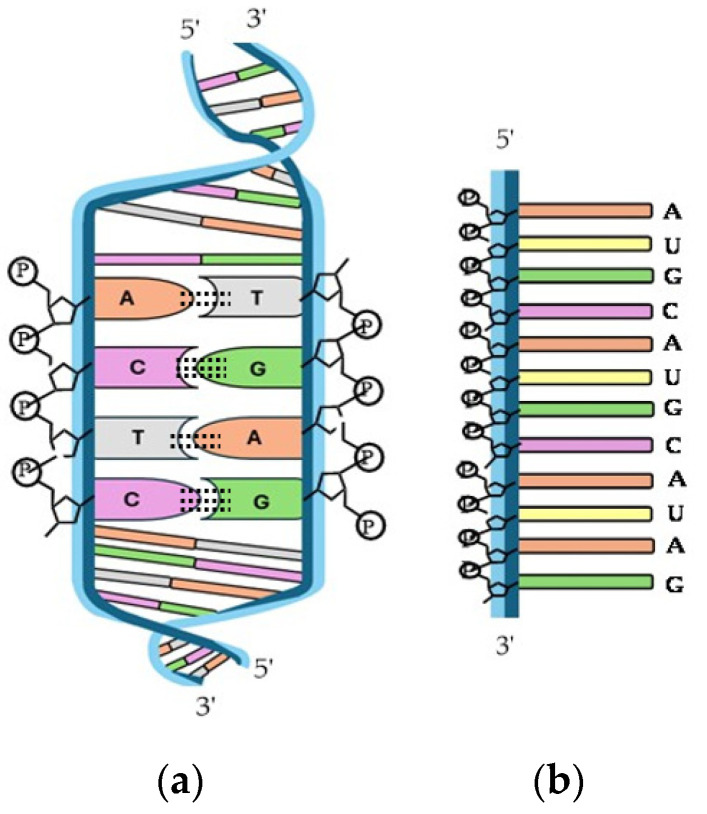
Structure of (**a**) double-helix DNA and (**b**) single-stranded RNA.

Nucleic acids are essential biological macromolecules that store and transmit genetic information, play a crucial role in protein synthesis, and are also involved in regulating gene expression and activating specific genes. These functions are fundamental to the development, machinery, and reproduction of all living organisms. There are two main types of nucleic acids: RNA and DNA (Figure 10). In 1953, Watson and Crick (WC) [303] proposed the three-dimensional model of DNA structure, which consists of two strands of nucleotides that wind together to form a double helix, whereas RNA is single-stranded. The base pairing in DNA involves the nitrogenous base A always combined with T, and C with G. This specific pairing is governed by the formation of hydrogen bonds between complementary bases: A-T forms two hydrogen bonds and C-G forms three hydrogen bonds. These hydrogen bonds play a critical role in the stability and specificity of double-stranded DNA.

DNA and RNA derivatives are known to be able to adsorb onto HAp [303]. In fact, like in the case of proteins, some of the earliest studies on the interaction between HAp and (poly)nucleotides arose from the utilization of inorganic matrices in chromatography [304]. Soon, the efficiency of HAp was discovered to be remarkable as a result of the high affinity for phosphate groups in the nucleotides [305,306,307], allowing even fine separations of double-stranded DNA, single-stranded DNA, and RNA [308].

One of the first applications of the nucleic acid condensation induced by CaP was its use by Graham and Van Der Eb for carrying and delivering adenovirus 5 DNA into KB cells [309]. Studies on HAp particles as vectors for DNA delivery in gene therapy have recently been reviewed by Zhu et al. [310].

Baglioni et al. [311] studied the interactions between DNA and CaPs in the search for composites to be used in nanomedicine. Inorganic carriers play a crucial role in enhancing cell adhesion and facilitating the entry of therapeutic materials into cells. In addition, and following the same idea, Drouet’s research group evaluated the adsorption of DNA on HAp, verifying the strong binding affinity between DNA and HAp. They also studied the desorption process triggered when there is an excess of phosphate ions in the medium by the competition of free phosphates with the DNA–phosphate backbone [312].

Yazaki et al. used lipids to provoke the precipitation of DNA onto the HAp surface [313,314], which has been demonstrated to enhance the gene expression of mesenchymal stem genes [315]. Furthermore, studies conducted by Gibbs et al. [316] supported a model of prebiotic polynucleotide synthesis in which a mineral, such as HAp, with anion exchange properties immobilizes high-molecular-weight products of a template-directed reaction. This avoids the spontaneous hydrolyzation processes that break the oligonucleotides in solution and are incompatible with the survival of long preformed oligomers. These authors observed that HAp adsorbs polyuridylic acid (poly(U)) from aqueous solution almost completely, without reducing its ability to function as a template for the synthesis of oligoadenylates. The adenine nitrogenous base (monomeric A) was found not to bind HAp directly; however, the functionalized HAp/poly(U) hybrid was found to incorporate 74% free adenine from the solution. The presence of HAp in the prebiotic environment could have led to the stabilization of nucleotides on its surface and made possible the further accretion of monomers to form polynucleotides. In this sense, it must be considered that two factors, an increase in the molecular weight and the secondary structure of the oligonucleotide, govern the HAp/polynucleotide binding. Regarding the secondary structure, it has been known for a long time that the linkage of double-stranded nucleotides and HAp is stronger than the one with single-stranded polynucleotides. Triple-stranded is even stronger than double-stranded, and quadruplexes even more so [317,318]. In the case of DNA, the negatively charged polyanion can be stabilized by positive charges, like Ca^2+^ ions in HAp, suggesting that DNA sequences will have an affinity for the crystalline mineral. The adsorption affinity constant, K_aff_, may change as the secondary structure is modified by increasing ionic strength. It has been shown that aptamer sequences with G-quadruplex structures have a higher affinity for apatite, relative to other sequences, when higher ionic strength is used to stabilize the G-quadruplex [319].

The use of DNA aptamers obtained by the process known as SELEX (Systematic Evolution of Ligands by EXponential enrichment) bonded to calcium phosphate materials has recently been studied. Aptamers are single-stranded oligonucleotides, typically ranging from 15 to 100 nucleotides in length [320]. Experimental and computational studies have shown that DNA aptamers are involved in the mineralization process and act as crucial affinity reagents or markers, enabling the differentiation between amorphous and crystalline materials. Duffy et al. [321] selected three aptamers (with a higher percentage of G nucleotides) and checked their affinity for HAp, ACP, and β-TCP. Large and similar K_aff_ values and binding at low aptamer concentrations (~50 nM) indicated the strong affinity of three aptamers for HAp (Table 6). The aptamers showed high selectivity for crystalline HAp over ACP and TCP. Kinetic analysis of the aptamers revealed a higher forward rate constant (k_f_) for aptamer 1, with the most compact G-quadruplex secondary structure.

### 3.4. HAp–Nucleic Acid–Metal Complex Hybrids

Metal ions play various roles in nucleic acid chemistry [287] and can be categorized as follows:

1. **Natural counterions:** Common cellular ions like K⁺, Mg^2^⁺, and Na⁺ act to neutralize the charges of polyanionic nucleic acids.
2. **Folding and stabilization:** These are essential for the proper folding of nucleic acids and for the stabilization of many RNA structures, as well as for the catalytic function of ribozymes, maintaining DNA structures such as Gua-quartets in telomeres and in Holliday junctions, and for the cross-shape structures formed during genetic recombination.
3. **Exogenous ions and mimicry:** Both physiological and non-physiological (exogenous) metal ions can mimic natural ions, affecting nucleic acid stability and charge neutralization and potentially causing DNA condensation or mutations.
4. **Damage by Reactive Oxygen Species (ROS):** Redox-active metal ions can cause damage to nucleic acids by breaking DNA strands. These ions can be essential (e.g., Cu⁺, Fe^2^⁺), both in certain disease states and in therapeutic and DNA sequencing applications.
5. **Phosphodiester reactions:** Metal ions are involved in the formation and degradation of nucleic acid phosphodiester bonds. They can provide the OH⁻ nucleophile, polarize P-O bonds, or stabilize transition states or leaving groups.

Nucleobases in the structure of nucleic acids are typically uncharged within the physiological pH range (4 < pH < 9). Metal coordination, like other chemical modifications of nucleobases, alters their acid–base properties and causes changes in pK_a_ values. The binding of metals to nucleic bases has been studied by several authors [322,323,324,325,326]. Studies by Sigel demonstrate that the protons of endocyclic NH groups become more acidic when a metal ion binds to an available nitrogen atom in the nucleobase ring. This phenomenon leads to changes in pK_a_ values, which can shift in either direction as the metal ion displaces a proton from the nucleobase and relocates it within the heterocycle, thereby generating a metal-stabilized rare tautomer. Metal–nucleobase bond formation occurs through the following contributions: (a) electrostatic interaction: between a metal ion and the nucleobase dipole, dominant in the gas phase but influenced by solvent polarity in solution or solid states; (b) hydrogen bond formation: between a metal’s coligands and nucleobase, contributing to the stability of the complex; and (c) polarization and charge transfer effects: involving electron redistribution within the metal–nucleobase complex, less influenced by solvents and counterions. As expected, the substitution of nucleobase protons with metal ions increases this contribution compared to the binding of the metal to an available lone pair of a ring nitrogen atom or an exocyclic oxygen.

The discussion will focus on guanine, which has a huge dipole moment and favorable orientation in the isolated base, resulting in high affinity of metal ions for the N7 position. In double-helical DNA, the affinity of metal ions for G-N7 is influenced by the nature of the base at the 5′ side, thus depending on the molecular electrostatic potential and N7 accessibility. Additionally, within nucleic acids, the affinity for metal ions is higher for DNA G-N7 than for RNA G-N7 [289]. Platinated DNA fragments confirm that guanine residues become more acidic after Pt coordination at N7 [327,328,329]. Conversely, the dipole moment and orientation of adenine make it less favorable for metal binding at N7, compounded by the steric hindrance of the exocyclic amino group.

**Figure 11:**
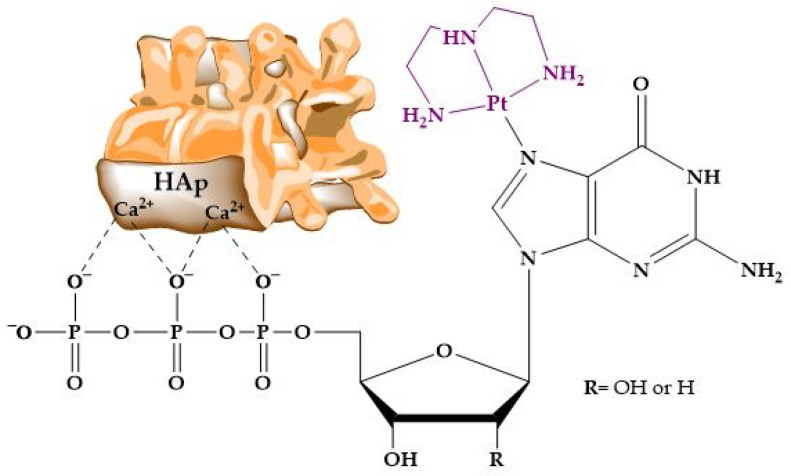
Structure and interaction of the platinum complex with the apatite surface.

As has been indicated before, HAp has been extensively used in column chromatography, and the literature covers multiple examples of this technical approach. However, as far as we are aware, the way functionalized HAp–nucleic acid hybrids engage with metal ions and coordination compounds has been investigated little. In one of the few instances, Benedetti et al. [302] focused their study on the adsorption of platinated nucleotide analogs onto HAp. The adsorption of these nucleotide complexes occurs through electrostatic interactions between the negative phosphate groups and positive ions on the surface of hydroxyapatite crystals (Figure 11). But the mere presence of phosphates does not ensure the adsorption of the nucleotide to HAp. The adsorption efficiency of these nucleotide derivatives was found to be influenced by the overall electrical charge of the metal complex. Platinated compounds with a net negative charge exhibited significant chemical adsorption onto the HAp surface, which could be reversed by acidification. In contrast, complexes with cationic or neutral charges showed very low adsorptions.

On the other hand, guanosine 5′-triphosphate (GTP) was anchored to the surface of Zn-substituted HAp nanoparticles, and its activity against osteosarcoma cells (Saos-2) was evaluated [330]. The functionalized nanoparticles induced differentiation into normal osteoblast cells in Saos-2 and provoked an enhancement of intracellular GTP content.

In other work, luminescent HAp nanoparticles containing Eu^3+^ ions were prepared by amidation of previously inserted AMP and final functionalization of the nanorods with poly(ethylene glycol) methacrylate (PEGMA) [331].

## 4. Perspectives and Conclusions

Despite the vast amount of knowledge acquired over the course of decades, many aspects of the complex world of HAp/protein and HAp/nucleic acid hybrids remain unclear. Although it may seem paradoxical, the early stages of HAp formation, from the first nuclei to the appearance of amorphous conglomerates, exhibit a high degree of uncertainty. Notwithstanding this, these first steps in CaP formation, prior to the differentiation into CaP species, are crucial for linkage to biomolecules, as has been discussed in the previous sections. Of course, the interaction between biomolecules and HAp itself, when the systems become complex, is far from being well understood, as is the case of ternary metal-doped HAp–biomolecules, in particular in the field of nucleotides, where extensive areas remain unaddressed and unexplored.

Moreover, new challenges in the attainment of tailored materials will have to be tackled, such as those related to the use of HAp matrices as drug delivery systems. Carriers do not only have a role in transport; they can also establish synergies with third chemical and biological parties, such as non-HAp-bonded biomolecules or cell structures, to improve the therapeutic response. In this sense, the incorporation of different metal ions and coordination complexes opens an infinite range of possibilities, for instance, those related to the preparation of new hybrid HAp/biomolecule multifunctional materials. This field has been explored for years, giving rise to interesting materials with creative combinations of properties, such as photocatalysts or magnetic systems exhibiting interesting biological properties [332,333,334,335,336,337,338]. However, as far as we are aware, relatively few quaternary HAp/metal/linker/biomolecule systems have been reported in the literature, which has mainly dealt with protein derivatives [339]. One of the most promising and yet seminal fields is the attainment of HAp-based protein- or nucleic acid-containing theragnostics. In this regard, it is worth mentioning the work reported by Iqbal, Kong, et al. [340]. These authors synthesized magnetic Fe_3_O_4_-HAp nanorods mixed with a non-ionic copolymer surfactant, Pluronic^®^F-127, using hydrothermal methods. In parallel, they encapsulated silk fibroin protein with a tri-block copolymer and, finally, they added chlorine e6, a photosensitizer, to build a platform available for photodynamic therapy. Certainly, previous studies were performed in analogous and simpler systems without biomolecules, like the F, Yb, and Ho-codoped HAp compound studied for bone repair and multimodal tracking by X-ray micro-computed tomography (micro-CT) images and upconversion fluorescence [341].

Self-healing biomaterials for bone regeneration that incorporate peptide-based nanocomposite scaffolds or hydrogels containing HAp mixed with polymers have been profusely studied [342,343,344,345]. However, to the best of our knowledge, the field of nucleic acid-driven self-healing HAp biomaterials remains unexplored, and this is paradoxical given that the internalization of HAp nanoparticles through clathrin-mediated endocytosis, which makes possible the approach to genetic material in the cell nucleus, is well established [346,347,348].

Although yet unmentioned, scientific efforts have focused on the application of the knowledge on HAp, bones, and teeth in the fields of archeology and forensic sciences. The presence of carbonate in bioapatite allows for the interpretation of the thermal history of a bone in terms of changes in its composition (P/C ratio and P/H ratio at higher temperatures), crystallinity, and crystal CaP phases formed. The gathered information can be useful for elucidating the maximum temperature reached by the bone and the environment. However, relevant aspects, as the speciation of the organic radicals formed and the concomitance of diagenetic processes which metal ions present in soils can influence, remain unclear [349]. Another Story, with capital letters, is that involved in the origin of Life on Earth, or anywhere in the Universe, from an astrobiological point of view. The role of minerals, such as clays, borates, or HAp itself, in the processes that led to the emergence of life will surely provide interesting findings in the next years. In particular, it will prove useful for studies on the interaction of inorganic surfaces, metal ions, and other chemical species, like water, with RNA as the macromolecules that, in prebiotic conditions, catapulted the passage from non-living to living beings (the so-called RNA world hypothesis) [350,351,352,353,354].

It must be highlighted that the role of exogenous metal ions and metal complexes in all the areas mentioned above is far from being well explored.

Finally, the present review has mainly focused on the chemical formulations of HAp hybrid compounds. However, no mention has been made of the instrumental techniques used to confirm the control of the composition, structure, size, and shape of the particles. This is not always an easy task, especially in those derivatives whose fast kinetics, instability, amorphous character, colloidal texture, and/or chemical complexity make a high degree of certainty on their characterization difficult. Excellent critical reviews have been published on this subject. Here, we only cite a handful of recently reported ones [355,356,357,358,359,360].

In summary, the non-toxic, stable, hard, biocompatible, and versatile compound called hydroxyapatite, specifically its interaction with living structures, is still a mystery, and its nature, mechanisms, relevance, and scientific and social impacts are yet to be unveiled. In light of this, research on the incorporation of metal complexes into hydroxyapatite/biomolecule hybrids is still at an early stage.

## Figures and Tables

**Table 1:** Selected calcium phosphate phases and structural details.

Name (Abbreviation) Formula	Ca/P Ratio	System (Space Group) Cell Parameters (Å, ⁰)	Refs.
Monocalcium phosphate anhydrous (MCPA) Ca(H_2_PO_4_)_2_	0.50	Triclinic (P1) a = 7.5577(5), b = 8.2531(6), c = 5.5504(3) α = 109.87(1), β = 93.68(1), γ = 109.15(1)	[9]
Monocalcium phosphate monohydrate (MCPM) Ca(H_2_PO_4_)_2_∙H_2_O	0.50	Triclinic (P1) a = 5.6261(5), b = 11.889(2), c = 6.4731(8) α = 98.633(6), β = 118.262(6), γ = 83.344(6)	[10]
Dicalcium phosphate anhydrous (DCPA, monetite) CaHPO_4_	1.00	$\mathrm{Triclinic}\;(P\bar{1}$) a = 6.90(1), b = 6.65(1), c = 7.00(1) α = 96.35(2), β = 103.90(2), γ = 88.73(2)	[11]
Dicalcium phosphate dihydrate (DCPD, brushite) CaHPO_4_∙2H_2_O	1.00	Monoclinic (Ia) a = 5.812(2), b = 15.180(3), c = 6.239(2) β = 116.42(2)	[12]
Amorphous calcium phosphates (ACPs) ^1^ Ca_x_H_y_(PO_4_)_z_∙nH_2_O (n = 3.0–4.5) ^2^	1.20–2.20 ^3^		
Octacalcium phosphate (OCP) Ca_8_H_2_(PO_4_)_6_∙5H_2_O	1.33	$\mathrm{Triclinic}\;(P\bar{1}$) a = 19.692(4), b = 9.523(2), c = 6.835(2) α = 90.15(2), β = 92.54(2), γ = 108.65(2)	[13,14]
α-tricalcium phosphate (α-TCP) α-Ca_3_(PO_4_)_2_	1.50	Monoclinic (P2_1_/a) a = 12.887(2), b = 27.280(4), c = 15.219(2) β = 126.20(1)	[15]
β-tricalcium phosphate (β-TCP, synthetic whitlockite) β-Ca_3_(PO_4_)_2_	1.50	Rhombohedral (R3c) (hexagonal setting) a = b = 10.439(1), c = 37.375(6)	[16]
Hydroxyapatite (HAp) Ca_10_(OH)_2_(PO_4_)_6_	1.67	Hexagonal (P6_3_/m) a = b = 9.424(4), c = 6.879(4)	[17]
Hydroxyapatite (HApM) M-Ca_10_(OH)_2_(PO_4_)_6_	1.67	Monoclinic (P2_1_/b) a = 9.419(3), b = 18.848(6), c = 6.884(2) β = 119.98(2)	[18]
Oxyapatite (OAp, voelckerite) ^4^ Ca_10_O(PO_4_)_6_	1.67	$\mathrm{Hexagonal}\;(P\bar{6}$) a = b = 9.432, c = 6.881	[19]
Tetracalcium phosphate (TTCP, hilgenstockite) Ca_4_O(PO_4_)_2_	2.00	Monoclinic (P2_1_) a = 7.023(1), b = 11.986(4), c = 9.473(2) β = 90.90(1)	[20]
Dicalcium diphosphate dihydrate (DCDD) Ca_2_(P_2_O_7_)∙2H_2_O	1.00	$\mathrm{Triclinic}\;(P\bar{1}$) a = 7.365(4), b = 8.287(4), c = 6.691(4) α = 102.96(1), β = 72.73(1), γ = 95.01(1)	[21]

^1^ ACPs are probably metastable amorphous states of different crystalline calcium phosphates. Roughly speaking, a dozen phosphate phases could exist in an amorphous state (e.g., amorphous HAp, amorphous TCP, etc.). An excellent review about them was published by Dorozhkin [22]. ^2^ The amount of water varies between 15 and 20%. ^3^ The range is wider, but the majority exhibit a Ca/P value close to 1.5. This would be consistent with the presence of Posner’s clusters, common units in ACPs, with formula Ca_9_(PO_4_)_6_ [23], which would evolve into different crystalline phases through distinct experimental conditions. ^4^ The nature and crystal structure of this compound remains controversial. The crystallographic information given in the table comes from the work by Henning et al. An interesting historical background about the different proposals published up to date is covered by Bulina et al. [24].

**Table 2:** Selected affinity constants for AA/HAp hybrids [185,186] (*). Adapted with permission from [186]. © Copyright 2016, published by the Royal Society.

Peptides	Net Charge	K_aff_/L·mol^−1^
Asp	−1	4166
Glu	−1	3021
Ala	0	286
Phe	0	2439
Pro	0	574
Met	0	621
Gly	0	1714
Cys	0	664
Gln	0	670
Ser	0	901
Leu	0	2026
Tyr	0	3030
Lys	+1	877

* Higher affinity constants have the greatest effect on crystal growth kinetics in refs [180,181,182,183,184,185,186].

**Table 3:** Experimentally determined binding affinities of HBPs. Adapted with permission from [219]. Copyright © 2020, American Chemical Society.

Peptides	Net Charge	K_aff_/L·mol^−1^
VTK	+1	2534
VTK_s	+1	2082
VTK_7E	0	1777
pVTK	−3	6194

**Table 4:** pK_a_ values for nucleic acid nitrogenous bases. Nitrogen acidic atoms are indicated.

Base	Atom	pK_a_
Uracil	N3	9.63
Thymine	N3	10.30
Guanine	N1	9.56
Guanine	N7	3.11
Cytosine	N3	4.60
Adenine	N1	4.10

**Table 5:** Comparison of pK_a_ values for several deoxy- and ribonucleotides as determined by potentiometric pH titrations in water at 25 °C and I = 0.1 M (NaNO_3_). Adapted with permission from [289]. Copyright © 2008 WILEY VCH Verlag GmbH & Co. KGaA, Weinheim.

Refs.	Acid NXP/dNXP	pK_a_ for N1H^+^ or N7H^+^ NXP/dNXP (4a)	pK_a_ for PO_2_(OH)^−^ NXP/dNXP (5a)	pK_a_ for N1H or N3H NXP/dNXP (6a)
[290,291]	H_2_(GMP)^±^/H_2_(dGMP)^±^	2.48 ± 0.04/2.69 ± 0.03	6.25 ± 0.02/6.29 ± 0.01	9.49 ± 0.02/9.56 ± 0.02
[289,290]	H_2_(AMP)^±^/H_2_(dAMP)^±^	3.84 ± 0.02/3.97 ± 0.02	6.21 ± 0.01/6.27 ± 0.04	
[292,293]	H_2_(CMP)^±^/H_2_(dCMP)^±^	4.33 ± 0.04/4.46 ± 0.01	6.19 ± 0.02/6.24 ± 0.01	
[293]	H(UMP)^−^/H(dTMP)		6.15 ± 0.01/6.36 ± 0.01	9.45 ± 0.02/9.90 ± 0.03
[289,294]	H_2_(GDP)^−^/H_2_(dGDP)^−^	2.67 ± 0.02/2.91 ± 0.07	6.38 ± 0.01/6.46 ± 0.03	9.56 ± 0.03/9.64 ± 0.04
[289,295]	H_2_(ADP)^−^/H_2_(dADP)^−^	3.92 ± 0.02/4.00 ± 0.03	6.40 ± 0.01/6.45 ± 0.01	
[296]	H_3_(CDP)^±^		6.39 ± 0.02/	
[296]	H_2_(UDP)^−^/H_2_(dTDP)		6.38 ± 0.02/6.44 ± 0.01	9.47 ± 0.02/9.93 ± 0.02
[289,297]	H_2_(GTP)^2−^/H_2_(dGTP)^2−^	2.94 ± 0.02/3.16 ± 0.05	6.50 ± 0.02/6.64 ± 0.02	9.57 ± 0.02/9.66 ± 0.04
[289,297]	H_2_(ATP)^2−^/H_2_(dATP)^2−^	4.00 ± 0.01/4.14 ± 0.02	6.47 ± 0.01/6.62 ± 0.03	
[297,298]	H_2_(CTP)^2−^	4.55 ± 0.02	6.55 ± 0.02	
[297,298]	H_2_(UTP)^2−^/H_2_(dTTP)^2−^		6.45 ± 0.01/6.52 ± 0.02	9.57 ± 0.02/10.08 ± 0.05

**Table 6:** Sequence, Gibbs free energy, affinity, and kinetics of aptamers for binding to HAp (*). Adapted with permission from [321]. Copyright © 2020 Elsevier B.V.

Aptamer	Sequence	k_f_ (min^−1^)	K_aff_ (M^−1^)	ΔG (Kcal∙mol^−1^)
**1**	CAGGGCGCTACGGTATGTGTTGGGTCTGGCGTAGGGCTGGC	12 ± 2 × 10^5^	3 ± 1 × 10^6^	−7.37
**2**	GAGCGCGCTACGGTATGTGTTGCGTGTGGCGTAGCGGTGCG	6 ± 1 × 10^5^	7 ± 4 × 10^6^	−8.54
**3**	CAGCGCCCTACGCTATGTCTTGCGTCTCGCCTAGCGCTCGC	2 ± 2 × 10^5^	7 ± 4 × 10^6^	−7.99

* K_f_: kinetic rate constant; K_aff_: adsorption affinity constant; **Δ**G: Gibbs free energy of folding for aptamers.
